# Correlation of Clinical Features in Oral Submucous Fibrosis: A 9-Year Retrospective Study

**DOI:** 10.4314/ejhs.v32i1.15

**Published:** 2022-01

**Authors:** Sneha R Sharma, Samiksha Chavan, Freny R Karjodkar, Kaustubh Sansare, S Bharathi, Shivani Singh

**Affiliations:** 1 Department of Oral Medicine and Radiology, Pacific Dental College and Hospital, Udaipur, Rajasthan, India; 2 Department of Oral Medicine and Radiology, Nair Hospital Dental College, Mumbai, Maharashtra, India

**Keywords:** Potentially Malignant Disorder, Oral Submucous Fibrosis, Hyperpigmentation, Oral Ulcers, VAS score, Tongue Protrusion

## Abstract

**Background:**

Oral Submucous Fibrosis is a chronic debilitating disease and potentially malignant disorder of the oral cavity known in medical literature for a long time. The study aims to correlate the clinical staging of Oral Submucous Fibrosis with various clinical findings of the oral mucosa like hyperpigmentation, erosions, ulcerations, VAS score, tongue protrusion, and cheek flexibility.

**Methods:**

A retrospective observational study was undertaken and records of 1267 clinically diagnosed cases of OSMF were included in the study. Clinical grading was done as per criteria by Lai DR et al. The observations were tabulated and subjected to statistical analysis using SPSS software.

**Results:**

In our study, the incidence of hyperpigmentation, erosions, and ulcerations was higher in Group C OSMF. Visual analog scores in the range of 5–6 was noted in 40.9% of the total subjects, out of which 36.2% belonged to Group C OSMF. Tongue protrusion of less than 25 mm was seen in 10.65% of the subjects. Cheek flexibility of less than 0.6 cm was seen in 19.62% of the subjects, most of which belonged to Group D.

**Conclusion:**

In our study, we found an increase in the occurrence and severity of symptoms with an increase in grades of OSMF, but this increase was not observed to be consistent. Hence classification based on a single clinical entity cannot be sufficient and correlations to other clinical findings should be studied over a large population and a multi-tier classification could be proposed in the future.

## Introduction

In historical medicine, Shushrutha mentioned a condition, “vidari” in the category of mouth and throat diseases. He analyzed progressive narrowing of the oral cavity, change in color of the oral mucosa, and pain on eating. The symptoms he observed precisely fit the findings of Oral Submucous Fibrosis (OSMF) (1). OSMF is a chronic, potentially malignant condition of the oral mucosa, first described by Schwartz in 1952. He reported a case of “atrophica idiopathic tropica mucosae oris” prevalent among the Indians in East Africa. In India, the condition was first described by Lal and Joshi in 1953. OSMF is a chronic, progressive, scarring high-risk precancerous condition involving the oral mucosa, seen primarily on the Indian subcontinent and in South East Asia (2).

Global estimates of OSMF show confinement to Indians and Southeast Asians, with an overall Indian prevalence rate of approximately 0.2–0.5% (3). The carcinomatous transformation of OSMF to oral squamous cell carcinoma (OSCC) has been estimated to be 7–30 % (4). Capsaicin present in chilies and alkaloids seen in areca nuts were believed to act as soluble irritants which are initiating factors for a juxta-epithelial inflammatory reaction. This inflammation leads to burning sensation, vesiculation, and ulceration of the oral mucosa. The epithelium can become atrophic owing to numerous reasons which make it vulnerable to these irritants and causes improper vascular channel formation resulting in decreased vascularity. These subsurface changes bring about defective healing and scarification. The cumulative effects of the initiating and promoting factors lead to fibrosis, which is a hallmark of the condition. Progressive fibrosis leads to multiple outcomes including a reduction in mouth opening, restricted tongue mobility, and difficulty in cheek blowing (5). It may remain either stationary or turn out to be severe, leaving the individual physically, socially, and psychologically challenged.

This chronic disease, based on its severity and extent, leaves a person debilitated on various levels. The subsurface inflammation and atrophy of the epithelium cause a burning sensation that affects the basic function of eating. The patients alter their diet to avoid recurrent episodes of pain on the consumption of food, leading to deficiencies. The importance of normal movements of the tongue and flexibility of the cheek is underestimated till it is hampered. The effects on these can disturb the quality of life of a patient. Normal movements of the tongue and floor of the mouth are critical for deglutition and speech. The reduction in cheek flexibility can also be embarrassing on few occasions. For example, not being able to perform a simple job of blowing air in balloons for any event can be humiliating on a social front. Previously several studies have been done, which have graded the clinical stages of OSMF based on the interincisal mouth opening. As per the literature search, limited studies have been conducted to measure erosions, ulcerations, VAS score, tongue protrusion, and cheek flexibility and to correlate these findings with the existing clinical grading of OSMF. Few cases of OSMF associated with areas of melanotic patches and pigmentations on the buccal mucosa have been reported in the literature (6, 7).

This study intended to correlate the clinical staging of Oral Submucous Fibrosis as per Lai DR classification of mouth opening with various clinical findings of the oral mucosa such as hyperpigmentation, erosions, ulcerations, VAS score, tongue protrusion, and cheek flexibility. It is important to study this correlation and impart adequate knowledge of the same while counseling patients.

## Materials and Methods

An Institutional retrospective observational study was undertaken in the unit of Oral Medicine and all OSMF case records from January 2010 till January 2019 were included in the study. The sample size comprised of case records of 1267 patients who were clinically diagnosed with OSMF from January 2010 till January 2019. However, the power of the study obtained was 80% at a 95% Confidence Interval. The Study was approved by the institutional ethical committee (letter no: EC-113/DOMR- 34 ND/2019).

**Inclusion criteria**: All case records of OSMF patients who had reported to the oral medicine unit, were included the study.

**Exclusion criteria**: Incomplete case records without details of clinical features like hyperpigmentation, erosions, ulcerations, VAS score, tongue protrusion, and cheek flexibility.

The investigating examiner had more than 25 years of experience in Oral Medicine, particularly in handling cases of OSMF at the point of retrieval of the data. Given this vast experience, the examiner did not undergo calibration. To maintain standardization, only the measurements and clinical examination done by the same examiner were included in the study. The mouth opening was recorded in millimeters using a Vernier Caliper by measuring inter-incisal mouth opening from the mesio-incisal angle of the upper central incisor to the mesio-incisal angle of the lower central incisor. The following standard protocol was followed for the measurement of mouth opening for all cases of OSMF - Before starting with the measurements, the internal jaws of the caliper were approximated and it was ensured that there was no gap between the two. Also, it was ensured that the movable external jaw coincided at 0 mm before beginning the measurements.

**Figure 1:**
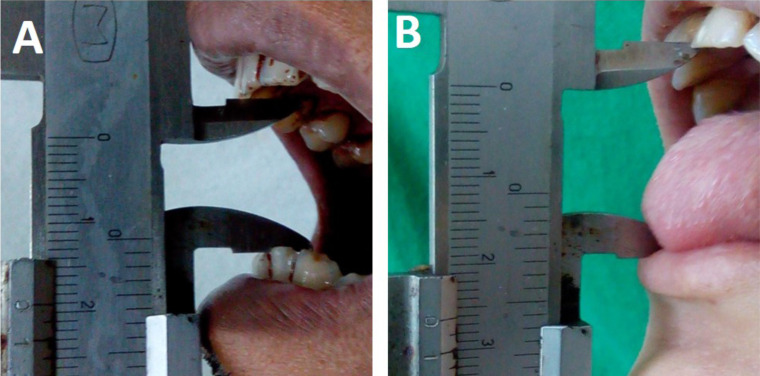
showing **(A)** Interincisal distance between the central incisors - 19 mm (Group D OSMF) and **(B)** Tongue protrusion measured from the incisal edge of maxillary central incisor to the tip of the tongue - 26 mm

The clinical criteria which were used for the diagnosis of cases of OSMF were based on the classification given by Lai DR (1995) based on the inter incisal distance (8) (Figure 1A).

Group A > 35 mm

Group B 30 — 35 mm

Group C 20 — 30 mm

Group D < 20mm

**Figure 2:**
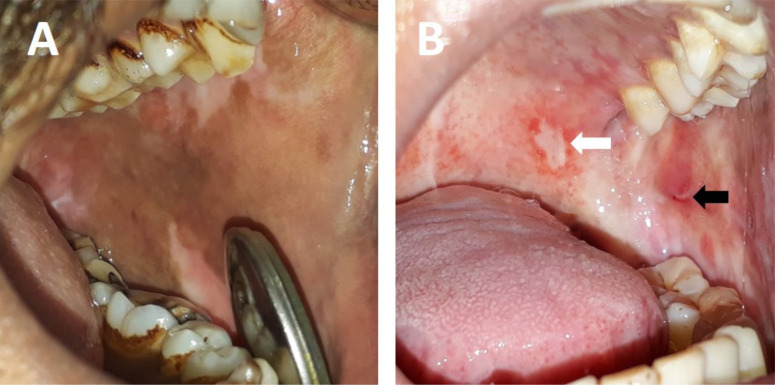
Left buccal mucosa showing **(A)** hyperpigmentated area and **(B)** erosion on the posterior aspect (black arrow) and ulceration on the posterolateral aspect of the junction hard and soft palate (white arrow)

Inspection of the mucosa was done for hyperpigmentation, erosions, and ulceration, and their presence or absence in the patient was noted (Figure 2). The severity and extent of the clinical features including VAS Score, tongue protrusion, and cheek flexibility were retrieved from the case records. A numeric rating Visual Analogue Scale (VAS) was used to determine burning sensation. The scale is graded on a 10-point scale from 0 to 10, where 0 indicated no burning sensation while 10 represented the worst burning sensation possible (9). Tongue protrusion was measured using a Vernier Caliper, from the normal mesio-incisal angle of upper central incisor to the tip of the tongue when maximally extended with mouth wide open (Figure 1B). Measurement of Cheek flexibility was done in accordance with the method by Mathur and Jha: Cheek flexibility = V1-V2. Two points were measured between at one-third the distance from the angle of the mouth on a line joining the tragus of the ear and the angle of the mouth (V2). The subject is then asked to blow his cheeks fully and the distance measured between the two points marked on the cheek (V1) (10).

**Statistical analysis**: All data obtained were entered in a Microsoft Excel sheet. Frequency distribution was done and Spearman's correlation test was performed to assess the correlation between the variables. The observations were tabulated and subjected to statistical analysis using SPSS software. (IBM Corp. Released 2012. IBM SPSS Statistics for Windows, Version 21.0. Armonk, NY: IBM Corp).

## Results

A total of 1267 patients met the inclusion criteria out of which 1120 were males and 147 were females. The patients were distributed into 4 age groups - 15 to 25 years (n = 161), 26 to 35 years (n = 557), 36 to 45 years (n = 395), 46 to 55 years (n = 154).

Based on mouth opening, a total of 353 (27.86%) patients were recorded with interincisal mouth opening ranging from 35.1–50 mm which were diagnosed as Group A OSMF (according to Lai DR Classification). A total of 239 (22.8%) patients were categorized into Group B OSMF. An inter-incisal mouth opening of 20.1–30 mm was recorded in a total of 464 (36.62%) patients suggestive of Group C OSMF and the remaining 211 (16.65%) patients showed an inter-incisal mouth opening of less than 25.1 mm indicating Group D OSMF (Table 1).

**Table 1:** Correlation between Mouth opening and OSMF group

	OSMF group				Total	R -value	P- value
	
Mouth opening	Group A	Group B	Group C	Group D			
Less than 20 mm	0	0	0	211	211	-.959	.000*
20 to 30 mm	0	0	464	0	464		
30.1 -35 mm	0	239	0	0	239		
More than 35 mm	353	0	0	0	353		
Total	353	239	464	211	1267		

A weak correlation was found with hyperpigmentation, erosions, and ulceration but these were statistically significant (p-value <0.05). A total of 27.07% of the participants showed the presence of hyperpigmentation of which maximum patients belonged to Group C. 34.01% of the population showed the presence of ulceration of which maximum patients belonged to Group C. The percentage of the population showing the presence of ulcers was 6.62% of which maximum belonged to Group B (31 patients) and Group C (30 patients) (Table 2).

**Table 2:** Correlation between Hyperpigmentation, Erosions and Ulcerations and OSMF group

Clinical Feature	Present / Absent	OSMF group				Total	R -value	P-value
		Group A	Group B	Group C	Group D			
Erosions	Absent	282	157	304	93	836	.224	0.000*
	Present	71	82	160	118	431		
Total		353	239	464	211	1267		
Ulcerations	Absent	341	208	434	200	1183	.015	0.000
	Present	12	31	30	11	84		
Total		353	239	464	211	1267		
Hyper- pigmentation	Absent	340	163	274	147	924	0.272	0.000*
	Present	13	76	190	64	343		
Total		353	239	464	211	1267		

A weak correlation was found with the VAS score and these correlations were statistically significant (p-value <0.05). Most of the patients recorded a VAS score of 5–6 which included 40.96% of the sample. Among these 188 patients belonged to group C (Table 3) based on tongue protrusion, the maximum number of patients showed a tongue protrusion in the ranges of 35.1–45 mm and 45.1–55 mm. A total of 22 participants showed tongue protrusion less than 15mm of which 12 subjects showed Group B OSMF (Lai DR et al) (Table 4).

**Table 3:** Correlation between Burning sensation (VAS) and OSMF group

		OSMF group				Total	R -value	P-value
		
		Group A	Group B	Group C	Group D			
Burning sensation	up to 2	14	8	15	2	39	.533	0.000*
	3–4	166	32	33	21	252		
	5–6	142	172	188	17	519		
	7–8	25	15	146	11	197		
	8–10	6	12	82	160	260		
Total		353	239	464	211	1267		

**Table 4:** Correlation between Tongue protrusion, cheek flexibility, and OSMF group

		OSMF group				Total	R -value	P-value
		
		Group A	Group B	Group C	Group D			
Tongue protrusion	up to	15 mm	5	12	3	2	22 -.582	0.000*
	15.1 – 25 mm	6	18	17	72	113		
	25.1 – 35 mm	15	23	120	119	277		
	35.1 – 45 mm	14	78	256	7	355		
	45.1 – 55 mm	229	104	57	5	395		
	55.1 – 65 mm	13	4	6	1	24		
	> 65mm	71	0	5	5	81		
Cheek flexibility	< 0.6	30	27	74	117	248	-.287	0.000*
	0.6 – 1	262	183	371	73	887		
	1–1.5	61	29	19	21	130		
Total		353	239	464	211	1267		

A weak negative statistically significant correlation was found with mouth opening, tongue protrusion, and cheek flexibility (p-value <0.05). A total of 248 patients showed cheek flexibility reduced to less than 0.6cm out of which maximum belonged to Group D (117 patients) (Table 4).

## Discussion

In this retrospective study, case records of 1267 subjects who were clinically diagnosed with OSMF were included. Dr. K. Ranganathan I *et al.*, carried out a study to set baseline data for mouth opening, cheek flexibility, and tongue protrusion. The average mouth opening for males was 47.5 mm and for females, it came out to be 44.6mm. Average cheek flexibility from the sample came out to be 9.7 mm in males and 9.0 mm in females. Average tongue protrusion in males was 24.9 mm and 24.8 mm in females (11).

Patil S. et al., in 2014 proposed a classification for OSMF based on cheek flexibility. This study included 412 patients presenting clinical signs and symptoms of OSMF. Cheek flexibility of 30 mm and above was present in 60.4% of the sample while cheek flexibility between 20–30 mm was noted in 29.6% of patients and in 10% patients, it measured less than 20 mm (12).

In our study, 19.62% of the sample showed cheek flexibility of less than 0.6 cm of which the maximum was from Group D and the least patients from Group B. Patil S. et al also accounted for vesicles and ulcers which were noted in 14.8% of the sample population (12). Our study reported ulceration in 6.62% of patients of which 2.44% of patients showed Group B OSMF (Lai DR). In a study done by Patil et al, 34.01% of the patients in the sample presented with erosions. Among these, the maximum were patients belonging to Group C OSMF. 431 patients showed erosions that were 34.07%, maximum belonged to Group C OSMF. One of the preliminary changes in the mucosa is the formation of vesicles which lead to erosion which persistently lead to ulceration. The sensation of burning on the mucosa is aggravated in all these stages.

L.B. Kumar, et al. conducted a clinicohistopathologic study to find the significance of burning sensation on clinical staging and histopathologic grading. The results of the study showed an increase in degranulated mast cells in cases with mild and moderate burning sensation and a subsequent decrease in these cells was seen in severe cases. Hence, suggesting that degranulated mast cells may be responsible for mild to a moderate burning sensation. In the present study, the burning sensation was recorded as a VAS score. The results showed 41% of the sample recorded burning sensation with a VAS score of 5–6 of which the maximum patients were in Group C. The highest VAS Score of 8–10 was more in Group D OSMF patients (13). Many theories have been discussed on the cause of burning sensation in an OSMF patient. Many factors including nutritive deficiency leading to anemia and microscopic changes owing to irritants and the release of mediators can be the cause of burning sensation during the disease process (14, 15).

Scrutinizing the results of our study, a trend was seen in the occurrence of hyperpigmentation, erosions, and ulceration which gradually increased till Group C patients and then reduced in Group D patients. In the case of VAS scores, each group showed maximum patients at a particular score, Group A in the range of 3–4, Group B and C in the range of 5–6, and Group D in the range of 8–10. In the case of tongue protrusion, the most severe condition in our study was less than 15 mm. The highest number of patients within this range belonged to Group B and not Group D as would have been expected. As seen in the data collected for cheek flexibility, it was noticed that maximum patients in Groups A, B, and C showed a reduction in cheek flexibility but the severe reduction of less than 0.6 cm was seen more in Group D patients.

Bearing in mind the wide range of clinical presentation at various stages of OSMF, while diagnosing a patient with OSMF it is imperative to examine the findings properly. The treatment of such an ailment should be a holistic approach as one cause may lead to a symptom which may be a risk factor for another physiologic derangement and the cycle is persistent. The limitation of our study included the lack of histopathologic confirmation for patients diagnosed with OSMF. The 4 groups divided according to Lai DR Classification, did not include an equal number of OSMF patients.

It can be concluded that to date various studies right from disease prevalence to classification and recently comparative studies for different treatment modalities have been undertaken. Clinical classifications have been proposed by many authors. Measurement of the inter-incisal opening being the most objective method has been in use for grading OSMF. In our study, we aimed at linking some of the other clinical findings with the existing classification. This could not only categorize patients more efficiently but also help educate the patients about the initial symptoms and changes associated with the disease. With this knowledge, the patients can report for treatment in the initial stages which can be helpful in early and more effective conservative management. In the future, a study with a bigger or multicentric sample can be carried out to find the correlation of hyperpigmentation, erosions, ulcerations, VAS score, tongue protrusion, and cheek flexibility. Based on this study a classification considering multiple parameters can be proposed which will help in the formulation of a specific and better treatment plan.
